# Prepyloric Gastric Ulceration Associated With Intermittent Nonsteroidal Anti-inflammatory Drug (NSAID) Use and Unintentional Exposure Through Herbal Supplements: A Case Report

**DOI:** 10.7759/cureus.114266

**Published:** 2026-08-10

**Authors:** José Serafio-Gómez, Christopher E Cárdenas Hernández, Diego Jasso Pasillas, Grecia N Pedraza, Amira S Roque Salado

**Affiliations:** 1 General Surgery, Secretaría de Salud del Estado de Aguascalientes, Aguascalientes, MEX; 2 College of Medicine, Universidad Cuauhtémoc, Campus Aguascalientes, Aguascalientes, MEX

**Keywords:** diclofenac, gastric, gastrointestinal damage, herbal supplementation, laparotomy, nsaid, perforation, prepyloric, supplement, ulceration

## Abstract

Perforated peptic ulcer (PPU) remains a common cause of an acute abdomen and a surgical emergency associated with significant morbidity and mortality when diagnosis and treatment are delayed. The primary established risk factor is the chronic use of nonsteroidal anti-inflammatory drugs (NSAIDs), alongside advanced age and a history of peptic ulcer disease. While the consumption of herbal supplements has increased worldwide and may cause mild gastric mucosal irritation, their role is minimal compared to the direct causal impact of NSAID exposure in severe gastrointestinal complications.

This report presents a case of a perforated prepyloric gastric ulcer linked to the chronic, unintentional consumption of undeclared NSAIDs in herbal supplements, such as Ortiga mas Ajo Rey (OAR). This case underscores the clinical necessity of obtaining an exhaustive medication history - including over-the-counter and alternative preparations - when evaluating patients with an acute abdomen, as patients may inadvertently ingest NSAIDs through unregulated herbal products.

A 60-year-old female patient with a history of well-controlled systemic hypertension presented with sudden-onset severe epigastric pain radiating to the right flank. The patient reported chronic NSAID use for several years, along with intermittent self-medication with a herbal supplement containing garlic and nettle. Chest radiography revealed free subdiaphragmatic air, highly suggestive of hollow viscus perforation. The patient underwent emergency exploratory laparotomy, which revealed a 2 cm perforated prepyloric ulcer (Johnson type III) with generalized peritonitis. Surgical management consisted of ulcer closure with continuous suturing and Graham patch placement, with an uneventful postoperative course and no immediate complications.

This case emphasizes that chronic NSAID exposure, including inadvertent exposure through adulterated over-the-counter herbal supplements containing undeclared pharmacologically active ingredients, represents the most plausible etiologic factor for peptic ulcer perforation in this patient. In contrast, the herbal components themselves cannot be considered causal based on the available evidence. A comprehensive medication history that specifically includes prescription drugs, over-the-counter NSAIDs, herbal supplements, and other nonprescription products is essential for identifying otherwise overlooked ulcerogenic risk factors. Early recognition of these exposures, together with prompt diagnosis and timely surgical intervention, remains critical to reducing morbidity and mortality in patients with PPU.

## Introduction

Acute abdomen syndrome (AAS) remains a common and potentially life-threatening clinical condition that frequently requires urgent surgical intervention. Among its multiple etiologies, perforation of a hollow viscus represents a true surgical emergency due to the risk of generalized peritonitis, sepsis, and death if not promptly recognized and treated. Delayed diagnosis and treatment are strongly associated with increased morbidity and mortality, making early recognition essential [1]. For this reason, the evaluation of patients presenting with acute abdomen requires a high index of clinical suspicion, supported by timely imaging studies, particularly in cases where clinical presentation is atypical or nonspecific. Perforated peptic ulcer (PPU) continues to be one of the most important causes of hollow viscus perforation requiring surgical management worldwide [1].

Peptic ulcer disease (PUD) is characterized by a defect in the gastric or duodenal mucosa caused by the action of gastric acid and pepsin, resulting from an imbalance between mucosal protective mechanisms and aggressive luminal factors [2]. Several risk factors have been consistently associated with peptic ulcer formation and complications, including advanced age, previous history of PUD, *Helicobacter pylori* infection, smoking, and particularly the chronic use of nonsteroidal anti-inflammatory drugs (NSAIDs) [2,3]. NSAID-induced mucosal injury occurs through inhibition of cyclooxygenase enzymes and reduced prostaglandin synthesis, leading to decreased mucosal blood flow, reduced bicarbonate and mucus secretion, and ultimately increased susceptibility to ulcer formation and perforation [2,3].

In recent years, the use of herbal and alternative medicine supplements has increased significantly worldwide, often without medical supervision [4,5]. Although these products are frequently perceived as safe due to their natural origin, Ortiga mas Ajo Rey (OAR) has been associated with gastrointestinal adverse effects [4,5]. Commonly used for their anti-inflammatory and cardiovascular properties, OAR may cause gastric mucosal irritation. Additionally, OAR exhibits antiplatelet activity, which could theoretically increase the risk of gastrointestinal bleeding, particularly in patients with concurrent exposure to established risk factors such as NSAIDs [4]. However, current evidence supporting a direct causal relationship between these supplements and peptic ulcer complications remains limited, and their role should be interpreted as potential rather than primary. Despite their widespread use, herbal supplements are often underreported during clinical evaluation, which may contribute to incomplete risk assessment in patients with gastrointestinal pathology [4].

We present the case of a 60-year-old female patient with acute abdominal pain and imaging findings compatible with hollow viscus perforation. This case highlights the importance of obtaining a comprehensive medication history, including herbal and over-the-counter product use, during the evaluation of patients presenting with AAS, as these agents may represent additional contributing factors alongside well-established etiologies such as NSAID use [1,4].

## Case presentation

A 60-year-old woman with a history of well-controlled systemic hypertension presented with acute abdominal pain. She reported chronic self-medication for approximately four years with OAR, taken twice daily without medical supervision. The patient denied a history of smoking, alcohol consumption, previous PUD, chronic proton pump inhibitor use, or prior upper gastrointestinal symptoms.

**Figure 1:**
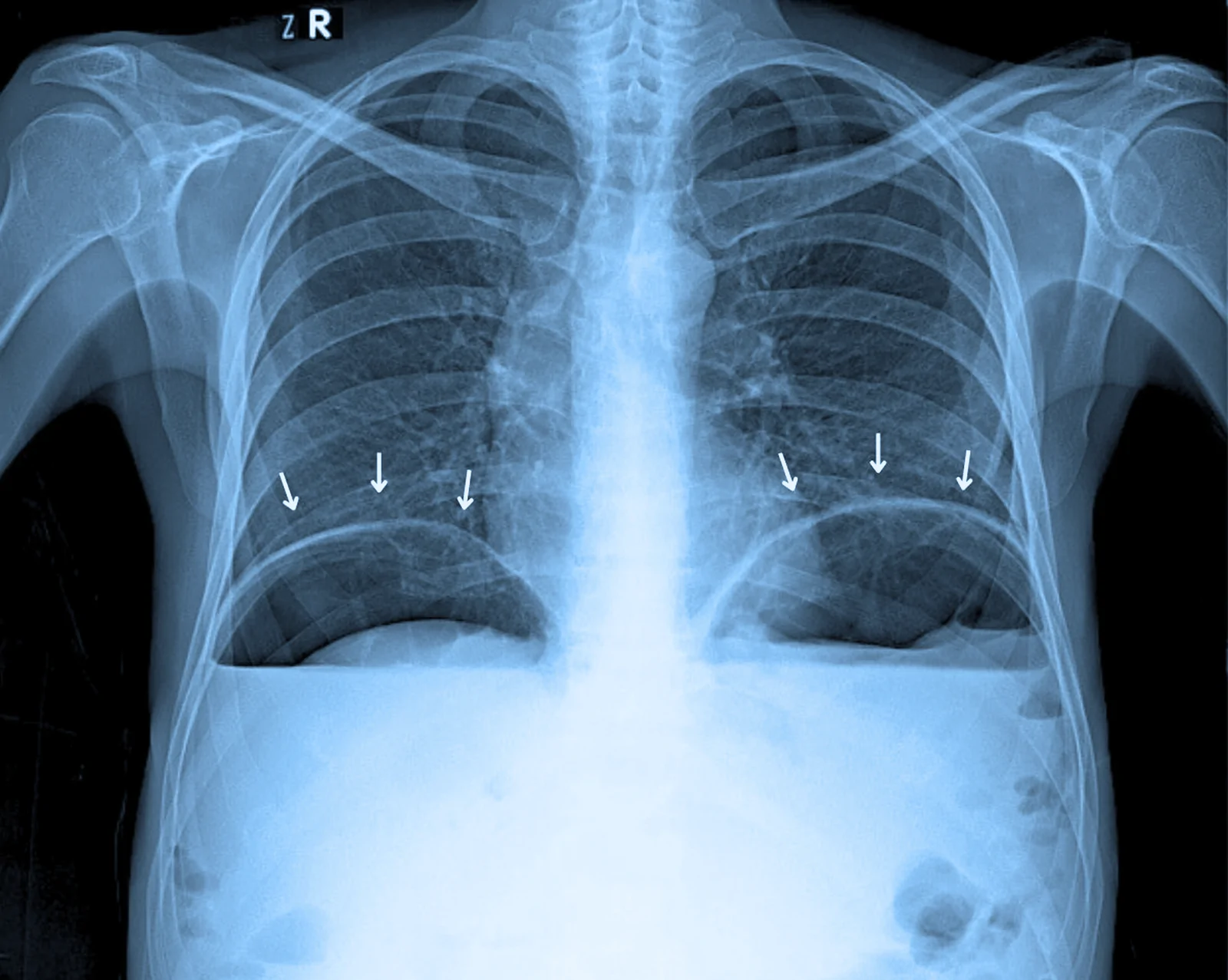
Posteroanterior (PA) chest X-ray with the presence of free subdiaphragmatic air consistent with pneumoperitoneum secondary to hollow viscus perforation. PA chest X-ray in the upright position showing the presence of free subdiaphragmatic air, evidenced by well-defined radiolucent areas beneath both hemidiaphragms (white arrows).

The patient presented to a private outpatient clinic with sudden-onset, severe epigastric pain radiating to the right flank. She reported recent intake of ketorolac 10 mg with minimal relief. Due to the persistence and severity of symptoms, a chest radiograph was obtained (Figure 1), which revealed free subdiaphragmatic air, highly suggestive of hollow viscus perforation. The patient was therefore immediately referred to a regional general hospital with a high clinical suspicion of perforated viscus.

Upon admission to the Emergency Department of the Regional General Hospital, physical examination revealed generalized abdominal pain, with an intensity of 10/10 on the visual analog scale (VAS) and clear signs of peritoneal irritation, including a positive Blumberg’s sign. The patient was hemodynamically stable.

Following the physical examination, the patient was managed with crystalloid solutions, and laboratory studies were requested, including a complete blood count (Table 1), coagulation profile (Table 2), and serum chemistry panel (Table 3).

**Table 1:** Hematological laboratory results of the patient.

Parameters	Results	Reference range
White blood cell count (WBC)	13.01 x 10^3^/µL	4.00-10.50 x 10^3^/µL
Red blood cell count (RBC)	5.13 x 10^6^/µL	4.10-5.30 x 10^6^/µL
Hemoglobin (Hb)	14.30 g/dL	12.20-16.80 g/dL
Hematocrit (Hct)	46.10%	36.60-50.40%
Platelet count	305 x 10^3^/µL	150-400 x 10^3^/µL
Lymphocytes	11%	20-50%
Segmented neutrophils	86%	40-75%
Blood group (Rh factor)	A (+)	-

**Table 2:** Coagulation profile results of the patient.

Parameters	Results	Reference range
Prothrombin time (PT)	12.2 seconds	12.5-16.0 seconds
Activated partial thromboplastin time (aPTT)	22.3 seconds	24.5-42.0 seconds
International normalized ratio (INR)	0.91	-

**Table 3:** Blood chemistry test results of the patient.

Parameters	Results	Reference range
Glucose	93 mg/dL	74.0-106.0 mg/dL
Blood urea nitrogen (BUN)	36 mg/dL	7.0-17.0 mg/dL
Urea	77 mg/dL	14.0-36.0 mg/dL
Serum creatinine	0.8 mg/dL	0.5-1.0 mg/dL
Uric acid	5.8 mg/dL	2.6-6.2 mg/dL
Lactate dehydrogenase	221 U/L	120.0-246.0 U/L
Alkaline phosphatase	86 U/L	38.0-126.0 U/L
Amylase	56 U/L	30.0-110.0 U/L
Lipase	79 U/L	23.0-300.0 U/L

**Figure 2:**
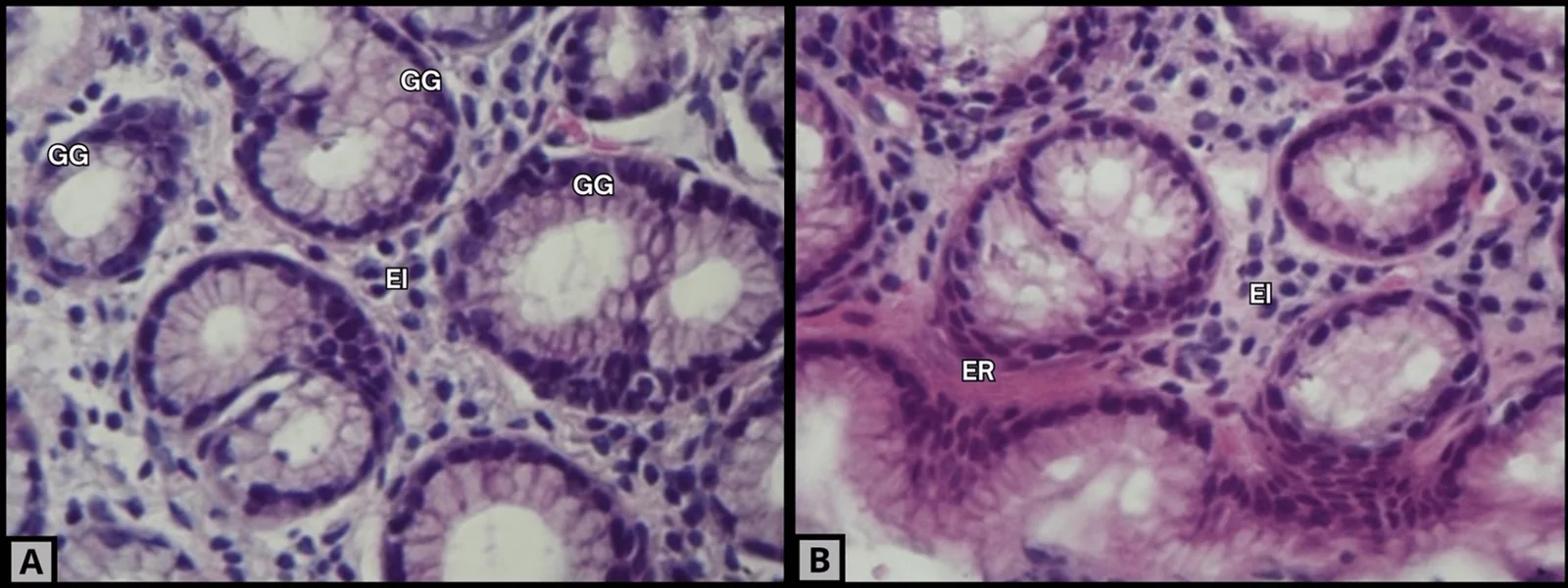
Representative hematoxylin-and-eosin-stained sections of the gastric biopsy. (A-B) Representative hematoxylin-and-eosin-stained sections of the gastric ulcer margins demonstrate a chronic inflammatory infiltrate (EI), predominantly composed of lymphocytes and plasma cells, within the lamina propria, along with regenerative epithelial changes (ER). The gastric glandular architecture (GG) is preserved, with no evidence of dysplasia or malignancy. No morphologic evidence of *Helicobacter pylori* organisms is identified in the examined sections.

After obtaining the laboratory results, the patient was taken to the operating room, where an exploratory laparotomy was performed due to a high suspicion of hollow viscus perforation. A supraumbilical midline incision was made, and dissection was carried out by planes until entry into the abdominal cavity, where gastrobiliary fluid was identified. The fluid was aspirated, and the cavity was dried until an approximately 2 cm ulcerative lesion was identified on the anterior surface of the prepyloric region along the greater curvature. Incisional biopsy specimens were obtained from the edges of the lesion for histopathological evaluation before definitive surgical management was undertaken (Figure 2).

Subsequently, the ulcer edges were debrided using cold dissection, and the perforation was closed with a continuous two-layer repair using 4-0 Prolene vascular suture (Ethicon, Raritan, NJ, USA). A Graham omental patch was then secured over the repair with 2-0 silk sutures. Hemostasis was confirmed, and a Penrose drain was placed through a separate right flank stab incision, with its tip positioned in the pelvic cavity. The abdominal fascia was closed with No. 1 Vicryl (Ethicon), the subcutaneous tissue with 3-0 Vicryl, and the skin with titanium staples. The Penrose drain was secured to the skin with 3-0 nylon, and the procedure was completed without intraoperative complications. Postoperative findings confirmed the diagnosis of a PPU Johnson type III with generalized peritonitis.

According to the modified Johnson classification, type I gastric ulcers are typically located on the lesser curvature of the gastric body. Type II ulcers are gastric body ulcers associated with a duodenal ulcer. Type III ulcers are located in the prepyloric region. Type IV ulcers occur on the proximal lesser curvature near the gastroesophageal junction. Type V ulcers can occur anywhere in the stomach and are associated with medication use [6].

The patient was managed with a postoperative dual antibiotic regimen. Over the following three days, supportive care with crystalloid solutions was continued. On the subsequent day, a soft diet was initiated after confirming the absence of leaks from the drainage system. The patient showed favorable clinical progression and was discharged on postoperative day 4.

## Discussion

PPU remains a high-severity surgical emergency, associated with substantial morbidity and mortality despite advances in medical and surgical management. Its annual incidence is estimated to range from 3.8 to 14 cases per 100,000 inhabitants, making it the second most common complication of PUD after upper gastrointestinal bleeding [7-9]. Although the overall incidence of uncomplicated PUD has declined steadily following the widespread use of proton pump inhibitors and systematic eradication of *H. pylori*, perforation rates have remained relatively stable, particularly among older adults [9].

The clinical presentation of PPU is heterogeneous and represents a major diagnostic challenge. While the classic presentation consists of sudden-onset severe epigastric pain accompanied by signs of peritoneal irritation, elderly patients may exhibit atypical or minimally expressive symptoms during the early stages, predisposing to diagnostic delays with adverse prognostic implications [9]. In the present case, the patient exhibited a typical acute surgical abdomen, characterized by abrupt severe epigastric pain, overt peritoneal signs, and radiographic evidence of subdiaphragmatic free air. Prompt recognition of these findings and immediate surgical referral likely contributed decisively to the favorable outcome, as diagnostic delays exceeding 24 hours are consistently associated with significantly increased mortality [9].

The laboratory findings were also consistent with this presentation. Leukocytosis with marked neutrophilia reflected the acute inflammatory response to generalized peritonitis, whereas the blood urea nitrogen (BUN) with preserved serum creatinine was suggestive of relative intravascular volume depletion secondary to the acute abdominal process. Although nonspecific, these abnormalities further supported the clinical diagnosis and the need for urgent surgical intervention [9].

From an anatomical and pathophysiological perspective, the modified Johnson classification remains a useful tool for characterizing gastric ulcers and guiding therapeutic decision-making. In this case, the lesion was classified as a type III gastric ulcer, defined by its prepyloric location within 3 cm of the pylorus and its association with gastric acid hypersecretion, sharing pathophysiologic features with duodenal ulcers [6]. These lesions, particularly when complicated, frequently require urgent surgical intervention and, in selected cases, more definitive strategies due to the risk of recurrence [9].

PUD arises from an imbalance between aggressive factors, primarily gastric acid and pepsin, and mucosal defense mechanisms, including mucus and bicarbonate secretion, mucosal blood flow, and prostaglandin synthesis [10,11]. Within this framework, exposure to NSAIDs remains one of the most well-established risk factors for mucosal injury and its complications. In the present case, chronic exposure to an over-the-counter supplement belonging to a product category for which the U.S. Food and Drug Administration (FDA) has issued safety alerts because of undeclared pharmacologically active ingredients, including diclofenac.

Diclofenac is an NSAID that inhibits cyclooxygenase-1 and -2 (COX-1 and COX-2), leading to reduced prostaglandin synthesis [7,12]. COX-1 inhibition compromises gastrointestinal mucosal protection by decreasing mucus and bicarbonate secretion, impairing blood flow, and delaying epithelial repair, thereby increasing susceptibility to injury. This mechanism is associated with serious gastrointestinal complications, including bleeding, ulceration, and perforation [7,13-15].

Importantly, safety alerts issued by the FDA have confirmed that supplements marketed as containing “Ortiga” may include undeclared pharmacologic agents such as diclofenac, dexamethasone, and methocarbamol [12,14]. These findings raise significant concerns regarding unintentional NSAID exposure, as well as the risk of additive toxicity and drug interactions. Among the potential contributors in this case, NSAID exposure, particularly from undeclared sources, should therefore be considered the primary driver of mucosal injury, whereas the role of the herbal components remains uncertain and likely secondary.

However, the specific supplement consumed by our patient was not chemically analyzed; therefore, the presence of undeclared diclofenac or other pharmacologically active ingredients could not be directly confirmed and remains an inference based on the available regulatory evidence.

Although several herbal components declared in the supplement, such as buchu, matarique, and shark cartilage, have been traditionally used for various medicinal purposes, current evidence supporting their efficacy or safety is limited [16-18]. Some have been associated with gastrointestinal adverse effects; however, a direct causal relationship with ulcer formation or perforation has not been clearly established. Their contribution in this case should therefore be regarded as speculative, and potentially limited to indirect or synergistic effects.

Collectively, the patient exhibited multiple risk factors for the development of PPU, including age greater than 60 years and chronic exposure to NSAIDs through a non-regulated supplement (Table 4) [7]. Although perforation has been reported even after short-term NSAID exposure, the presence of a 2 cm prepyloric ulcer in this case suggests underlying chronic mucosal injury rather than a de novo lesion developing within hours [7,15].

**Table 4:** Perforated peptic ulcer - risk factors. Adapted from Bielsa et al. [7], licensed under the Creative Commons Attribution (CC BY 4.0) license.

Risk factors
Advanced age (>60 years)
*Helicobacter pylori* infection
Chronic nonsteroidal anti-inflammatory drug (NSAID) use
Smoking
Alcohol consumption
Acid hypersecretion

This clinical pattern may be interpreted within a hypothetical “two-hit” model, in which chronically compromised mucosa due to long-standing exposures is subsequently destabilized by additional injury. However, this framework remains speculative and should not be interpreted as a confirmed pathogenic mechanism.

From a diagnostic standpoint, PPU is a surgical emergency whose prognosis depends directly on timely diagnosis and early operative management. Computed tomography is currently regarded as the most sensitive imaging modality for detecting pneumoperitoneum and localizing the site of perforation, clearly outperforming plain radiography [9,19,20]. Nevertheless, abdominal or chest radiography continues to play an initial role in many emergency settings despite its limited sensitivity and potential for false-negative results [9,19,20]. In the present case, early identification of subdiaphragmatic free air enabled rapid diagnostic confirmation and prompt surgical intervention, in accordance with current recommendations [9].

Overall, the available evidence supports NSAID exposure, particularly from undeclared sources, as the most plausible explanation for ulcer formation and subsequent perforation in this patient, while alternative explanations remain less substantiated. However, causality cannot be definitively established due to the absence of direct pharmacologic confirmation of the supplement contents at the time of exposure.

Furthermore, histopathological examination of incisional biopsy specimens obtained from the ulcer margins demonstrated chronic antral and oxyntic gastritis with mild inflammatory activity, erosion, and epithelial regeneration, without morphologic evidence of *H. pylori* infection, dysplasia, or malignancy. These findings substantially narrow the differential diagnosis by excluding the most common alternative etiologies of gastric ulceration requiring consideration in this clinical setting, particularly *H. pylori*-associated PUD and gastric carcinoma [3,7,9]. When interpreted together with the absence of other established risk factors, the close temporal relationship between initiation of the over-the-counter supplement and symptom onset, the well-established association between NSAID exposure and peptic ulcer perforation, and the FDA warnings regarding undeclared diclofenac in commercially available herbal supplements support exposure to an adulterated NSAID-containing supplement as the most plausible precipitating factor for ulcer formation and subsequent perforation in this patient [11-15]. Nevertheless, because chemical analysis of the ingested supplement was not performed, a definitive causal relationship cannot be established. Accordingly, this case should be interpreted as providing compelling circumstantial evidence rather than definitive proof of causality while underscoring the potential hazards associated with the use of unregulated dietary supplements containing undeclared pharmacologically active ingredients [12,14].

Finally, this case underscores the risks associated with indiscriminate use of unregulated supplements and highlights the importance of a thorough clinical history that actively explores the consumption of “herbal” or alternative products. It also reinforces the responsibility of healthcare professionals to educate patients and prescribe evidence-based therapies, with the aim of preventing potentially serious and avoidable complications.

## Conclusions

PPU remains a life-threatening surgical emergency requiring prompt recognition, accurate diagnosis, and timely operative management to prevent progression to generalized peritonitis, sepsis, and multiorgan failure. While NSAID exposure remains the principal driver of gastric mucosal injury, this case highlights the potential for inadvertent exposure to undeclared NSAIDs, particularly diclofenac, through unregulated over-the-counter herbal supplements. Histopathological examination of the ulcer margins demonstrated chronic gastritis with regenerative epithelial changes, without morphologic evidence of *H. pylori* infection, dysplasia, or malignancy, thereby strengthening the clinical inference that NSAID exposure represented the most plausible precipitating factor for ulcer formation and subsequent perforation. Although chemical analysis of the ingested supplement was not available and a definitive causal relationship cannot be established, the temporal association, exclusion of the principal alternative etiologies, and existing regulatory evidence of adulterated supplements support this interpretation. The favorable clinical outcome further emphasizes the importance of early imaging, prompt surgical intervention, and histopathological evaluation in the management of PPU. Finally, this case underscores the need for a comprehensive medication history that specifically includes over-the-counter and herbal supplements, as recognition of undeclared pharmacologically active ingredients may improve diagnostic accuracy, identify otherwise overlooked ulcerogenic risk factors, and ultimately reduce morbidity and mortality in patients presenting with an acute abdomen.
